# Cytokeratin-18 increase as a signal for early liver cell changes in high-fat diet-induced obesity before overt steatohepatitis

**DOI:** 10.3389/fendo.2026.1853417

**Published:** 2026-07-08

**Authors:** Özlem Özdemir, Çağrı Akalın, Fuat Ekiz, Pınar Naile Öğüten, Orhan Baş, Ahmet Bayrak, Yeliz Kaşko Arıcı

**Affiliations:** 1 Department of Internal Medicine, Medicine Faculty of Ordu University, Ordu, Türkiye; 2 Department of General Surgery, Medicine Faculty of Ordu University, Ordu, Türkiye; 3 Department of Gastroenterology. Medical Park Ordu Hospital, Ordu, Türkiye; 4 Department of Histology and Embryology. Medicine Faculty of Samsun University, Samsun, Türkiye; 5 Department of Anatomy, Medicine Faculty of Ordu University, Ordu, Türkiye; 6 Department of Medical Biochemistry, Medicine Faculty of Ordu University, Ordu, Türkiye; 7 Department of Biostatistics, Medicine Faculty of Ordu University, Ordu, Türkiye

**Keywords:** cytokeratin (CK) 18, liver cell, metabolic dysfunction associated fatty liver disease (MASLD), non alcoholic fatty liver disease (NAFLD), obesity

## Abstract

**Objective:**

The major cause of non-alcoholic fatty liver disease (NAFLD) is obesity; in fact, this condition has been defined as metabolic dysfunction-associated steatotic liver disease (MASLD). Non-invasive diagnosis and grading of NAFLD are the subjects of the studies. Cytokeratin-18 (CK-18) is particularly highlighted as an indicator of liver cell death and steatohepatitis, but its relationship with obesity remains unclear. This study aimed to evaluate the metabolic parameters, CK-18 levels, and liver histopathology in high-fat diet-induced obesity in rats.

**Methods:**

Rats were fed either a normal-fat diet (NFD) or a high-fat diet (HFD) for eight weeks. Body weights, metabolic-liver parameters and CK-18 levels were assessed at study end. The liver histopathological changes (steatosis, lobular inflammation, ballooning, fibrosis) and NAFLD activity score were examined.

**Results:**

Increased final body weight (335.8 ± 20.2 vs 291.5 ± 6.25 g; p<0.001), glucose (186.4 ± 46.9 vs 130.0 ± 16.2 mg/dL; p=0.011), insulin (4.30 ± 0.70 vs 2.23 ± 0.82 IU/L; p<0.001), HOMA-IR (2.03 ± 0.79 vs 0.73 ± 0.34; p=0.003), ALT (72.34 ± 12.35 vs 55.43 ± 5.75 U/L; p=0.009) and CK-18 (1.46 ± 0.18 vs 0.88 ± 0.18 ng/mL; p<0.001) levels and decreased AST/ALT ratio (1.16 ± 0.09 vs 1.32 ± 0.14; p=0.020) were observed in HFD group compared to NFD group. Histologically, all HFD specimens showed ballooning (8/8) and half showed steatosis (4/8). NAFLD score remained ≤3 in all specimens nevertheless CK-18 values tended to co-occur with higher ALT levels and ballooning formation within the HFD group.

**Conclusıon:**

HFD-induced obesity was associated with increased CK-18 and the hepatocellular changes as remarkable ballooning without the overt steatohepatitis. These findings support CK-18 interpreting as a signal for early hepatocellular injury in obesity.

## Graphical Abstract

**Figure:**
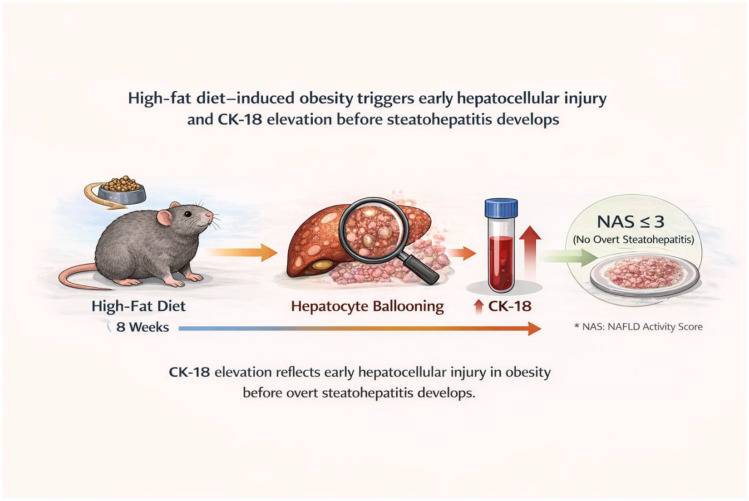

## 1 Introduction

Obesity and non-alcoholic fatty liver disease (NAFLD) are closely related metabolic disorders frequently experienced by physicians. Obesity is a growing problem worldwide; it has more than doubled since 1990 and in 2022, 43% of adults aged 18 years and over were overweight and 16% were living with obesity according to data of World Health Organization (1). A recent review analyzing 479 studies from 38 countries revealed a global prevalence of 30.2% for NAFLD (2). The prevalence of NAFLD increases with the increasing obesity prevalence and although it varies between regions, NAFLD prevalence varies from 60 to 95% in populations with obesity (3). Obesity, described as intracellular lipid accumulation and increased adipose tissue, is associated with dyslipidaemia and lipid deposition in liver tissue. According to a systematic review, the global prevalence of dyslipidemia range from 20% to 80% (4). Physical inactivity, unhealthy nutrition, lifestyle factors or other medical conditions lead to the development of dyslipidemia, obesity and NAFLD which are accepted as the major risk factors for the cardiovascular diseases (5). NAFLD has a histological spectrum ranging from simple liver steatosis as non-alcoholic fatty liver (NAFL) to steatosis with hepatocellular damage and inflammation described as non-alcoholic steatohepatitis (NASH). These pathological changes, including inflammation, necrosis and apoptosis, can progress to fibrosis, leading to cirrhosis and even hepatocellular carcinoma (6). NAFLD was redefined in 2020 by an international expert consensus as a metabolic-associated fatty liver disease (MAFLD) and then in 2023, the NAFLD Nomenclature Consensus group, led by the American Society of Hepatology, European Society of Hepatology, and Latin American Society of Hepatology, proposed a new name for NAFLD/NASH and classification of fatty liver disease as metabolic dysfunction-associated steatotic liver disease (MASLD)/metabolic dysfunction-associated steatohepatitis (MASH). The diagnosis was accepted if there is an evidence of hepatic steatosis by imaging, blood biomarkers/scores or histology, plus one of the three conditions: overweight/obesity or diabetes mellitus type 2 or the presence of metabolic risk abnormalities (7, 8).

Obesity and insulin resistance stimulate *de novo* lipogenesis with impaired lipolysis and loss of hepatic function. When lipid storage exceeds hepatic capacity, lipotoxicity begins and contribute to the development of the hepatocellular inflammation and apoptosis. The adipocyte expansion following the central deposition of fatty substrates is the main driver of NAFLD and related consequences (9). NAFLD is diagnosed using blood screening tests, imaging tests, and liver biopsy. There is no doubt that the biopsy for diagnosing NAFLD is definite method. But, it is an invasive procedure, so may not be acceptable by some patients, requires hospitalization, and has the risk of hemorrhage In addition, it does not provide a diagnosis in early hepatocellular changes and is poorly performed in obese patients (10). Therefore, the noninvasive biomarkers for diagnosis of NAFLD are valuable, and this is the fundamental reason why this study is conducted on rats. As NAFLD is currently the most common cause of abnormal liver function tests, the laboratory tests routinely used for the assessment of NAFLD are alanine aminotransferase (ALT) and aspartate aminotransferase (AST) (11). In recent years, lipid-related indices have been proposed useful for the NAFLD diagnosis. An independent association between triglycerides to high-density lipoprotein cholesterol ratio (TG/HDL-C) and NAFLD has been reported (12). Again, total cholesterol to high-density lipoprotein cholesterol ratio (TC/HDL-C) has been reported as a significant predictor of nonalcoholic fatty liver (13). However, the marker properties of these parameters are not precise. In recent years, surpassing these parameters, cytokeratin-18 (CK-18) is considered as a potential biomarker of NAFLD showing the inflammation and apoptosis in liver cells. CK-18 is an intermediate filament protein and major structural component of the cytoplasm in simple epithelial tissues such as liver, pancreas, intestines. The first descriptive studies on cytokeratin were conducted in the early 1980s. Cytokeratins were identified in epithelial cells, and their properties were described. As a member of the cytokeratin family, CK-18 is a structural protein that forms the epithelial tissue scaffold and protects the cell structure (14). In the 1990s, some researchers conducted a clinical pilot study validating the clinical utility of measuring serum Keratin-18 (K18). To investigate Keratin-18 (K18/CK18) for clinical liver disease, they generally used Tissue Polypeptide-Specific (TPS) antigen assays and caspase-cleaved fragments (M30, M65) as a marker for hepatocyte apoptosis and liver disease in steatohepatitis subsequently. When this scaffold is disrupted during cell injury, plasma-membrane blebs form and rupture, releasing keratin-18 fragments into the circulation (15). On this basis, Tarantino et al. examined the tissue polypeptide-specific antigen (TPS), a serological mirror of keratin 18, and reported that circulating TPS identified steatohepatitis with a sensitivity of 92% and a specificity of 96% and discriminated from simple fatty liver more accurately than alanine aminotransferase, ultrasonography, or their combination (16). These early pilot clinical studies first demonstrated the feasibility of circulating K18-related markers in NAFLD and helped lay the methodological groundwork for subsequent larger investigations. In the 2000s, the studies on the relationship between CK-18 and NAFLD accelerated, and numerous studies comparing it to various inflammation parameters were conducted. CK-18 was represented as a marker of hepatocyte apoptosis prominent in NASH and absent in simple steatosis (17).

CK-18 has been proposed as a biomarker that indicates the severe stage of NAFLD, especially the progressive disease as NASH. However, the development of NAFLD/MASLD is a process, and there is no clear consensus on the extent to which CK-18 levels are affected by the changes in liver cells in obesity. The aim of the present study was to evaluate circulating CK-18 levels together with liver histopathology and metabolic parameters in a high-fat diet induced obesity with the focus of NAFLD/MASLD.

## 2 Methods

### 2.1 Study design and dietary intervention

Twenty four-week-old male Sprague Dawley (SD) rats weighing 250–300 g (n =16) were obtained from Experimental Animals Application and Research Center of Samsun University, Samsun, Turke**y**. All rats were placed in 40x40 cm, 30 cm high cages to avoid restricting their movement. Each cage contained two rats. All rats were kept in an air-conditioned room with a 12 hours of light and dark cycle at constant temperature and humidity (23 ± 1°C and 55-65%, respectively) with free access to food and water. After one week of adaptation, rats were randomly divided into two groups (n =8) and fed on a normal-fat diet (NFD) and a high-fat diet (HFD). NFD was standard feed diet containing 10% kcal fat, 20% protein, and 70% carbohydrates. NFD primarily included corn, oats, and wheat, with added essential polyunsaturated fats such as soybean oil or corn oil. For the HFD group, we preferred the “cafeteria diet” enriched in saturated fats, cholesterol, and fructose to model metabolic syndrome and obesity. It contained 60% fat, 20% carbohydrates, and 20% protein. “Cafeteria” style diet consists processed, unhealthy, but delicious products such as chocolate, cake, cookies which induce hyperphagia and is used for causing metabolic syndrome in experimental obesity studies. Both NFD and HFD were obtained from Arden Research and Experiment Company, Ankara, Turkey. The rats were fed according to feeding guidelines for laboratory animals (18). We used a standard feed allocation of 10 grams per 100 grams of body weight. We planned a feed allocation of 30 grams per day for each rat. Considering the rats’ nocturnal eating habits and to eliminate the risk of malnutrition, we placed twice the required amount of feed daily. The rats’ weights were recorded on two week basis. Two rats of the NFD group died without showing any signs of disease in the 6th and 7th weeks of the study. They were at normal weights and continued their daily activities until death. They showed no signs of illness. There was no bleeding, dehydration, or ingestion of any toxic substance. At the end of the 8-week treatment period, all rats were sacrificed by cervical dislocation under deep anesthesia with intraperitoneal of ketamine 90 mg/kg (Ketalar; Eczacıbaşı, Istanbul, Türkiye) and xylazine hydrochloride 3 mg/kg (Rompun; Bayer, Leverkusen, Germany).

### 2.2 Ethical considerations

The animal experiments were conducted with the approval of the Animal Experiments Local Ethics Committee of Ordu University(16/02/2021 no: 82678388).

### 2.3 Biochemical analysis

It was entered from the bifurcation aorta with a 10 cc injector, 5 cc of blood were taken and transferred to appropriate laboratory parameter tubes with empty glass. Serum glucose, total cholesterol (CHOL), triglyceride (TG), high-density lipoprotein cholesterol (HDL), low-density lipoprotein cholesterol (LDL), and transaminases as Aspartat Aminotransferaz (AST), Alanin aminotransferaz (ALT) measurements were made using standard colorimetric methods (Otto Scientific Ankara, Turkey). Serum insulin and CK-18 measurements were made using an enzyme-linked immunosorbent assay (ELISA; Sunred, Shanghai, China). ELISA measurements were made twice and the result was determined by averaging the two measurements. Derived ratios were calculated as follows: AST/ALT, CHOL/HDL, and TG/HDL. HOMA-IR was calculated by the formula as “fasting glucose (mg/dL) × fasting insulin/405”. All the measurements were performed in Ordu University Biochemistry Research Laboratory.

### 2.4 Histopathological examination

Histopathological Examination was performed by a histology specialist in Pathology and Histology Research Laboratory of Ordu University. Rat liver specimens were fixed with 10% formaldehyde and embedded in paraffin. Thick paraffin sections (4 μm) were cut from each specimen. The obtained sections were stained with Hematoxylin-Eosin (H&E) and Gamori Trichrome stains to evaluate their parenchymal and stromal structures after being deparaffinized and rehydrated. Slides were viewed using a light microscope (Carl Zeiss, Primostar 3) and imaged with the camera (Zeiss Axicom 105 color) attached to the microscope. Histopathological scoring was performed according to the NAS scoring system developed by Kleiner et al. (19). According to this scoring system; steatosis (0–3), lobular inflammation (0–3), hepatocyte ballooning (0–2) and fibrosis (0–4) were identified. The presence of steatosis is necessary and only statosis formation is described as non-alcoholic fatty liver (NAFL). Histopathological status is described as “NAFLD” if the NAS score is 1–3 and described as NASH if the NAS score is ≥ 4. Fibrosis score is 0-4, score 0 is defined as “no signs of fibrosis” and score 4 is defined as “cirrhosis”.

### 2.5 Statistical analysis

All statistical analyses were performed using IBM SPSS Statistics (v28; IBM Corp., Armonk, NY, USA). Continuous variables were summarized as mean ± SD. HOMA-IR was calculated as fasting glucose (mg/dL) × fasting insulin/405. Normality was assessed using the Shapiro–Wilk test, and variance homogeneity was evaluated using Levene’s test. Continuous variables were compared between groups using the independent-samples t-test or Welch’s t-test, as appropriate. Steatosis, lobular inflammation, and fibrosis were analyzed as binary variables (absence [score=0] vs presence [score>0]) and compared using Fisher’s exact test. Ballooning scores were compared between groups using the exact Mann–Whitney U test. Effect sizes were reported where appropriate. Within the HFD group, CK-18 levels were compared between animals with ballooning score 1 and those with ballooning score 2 using the exact Mann–Whitney U test, with rank-biserial correlation reported as the effect size. The association between CK-18 and ballooning severity was additionally evaluated using Spearman’s rank correlation analysis. Graphical visualizations were generated using Minitab 20 and Python (Matplotlib). All tests were two-tailed, and p<0.05 was considered statistically significant.

## 3 Results

### 3.1 Body weights and biochemical results

**Figure 1:**
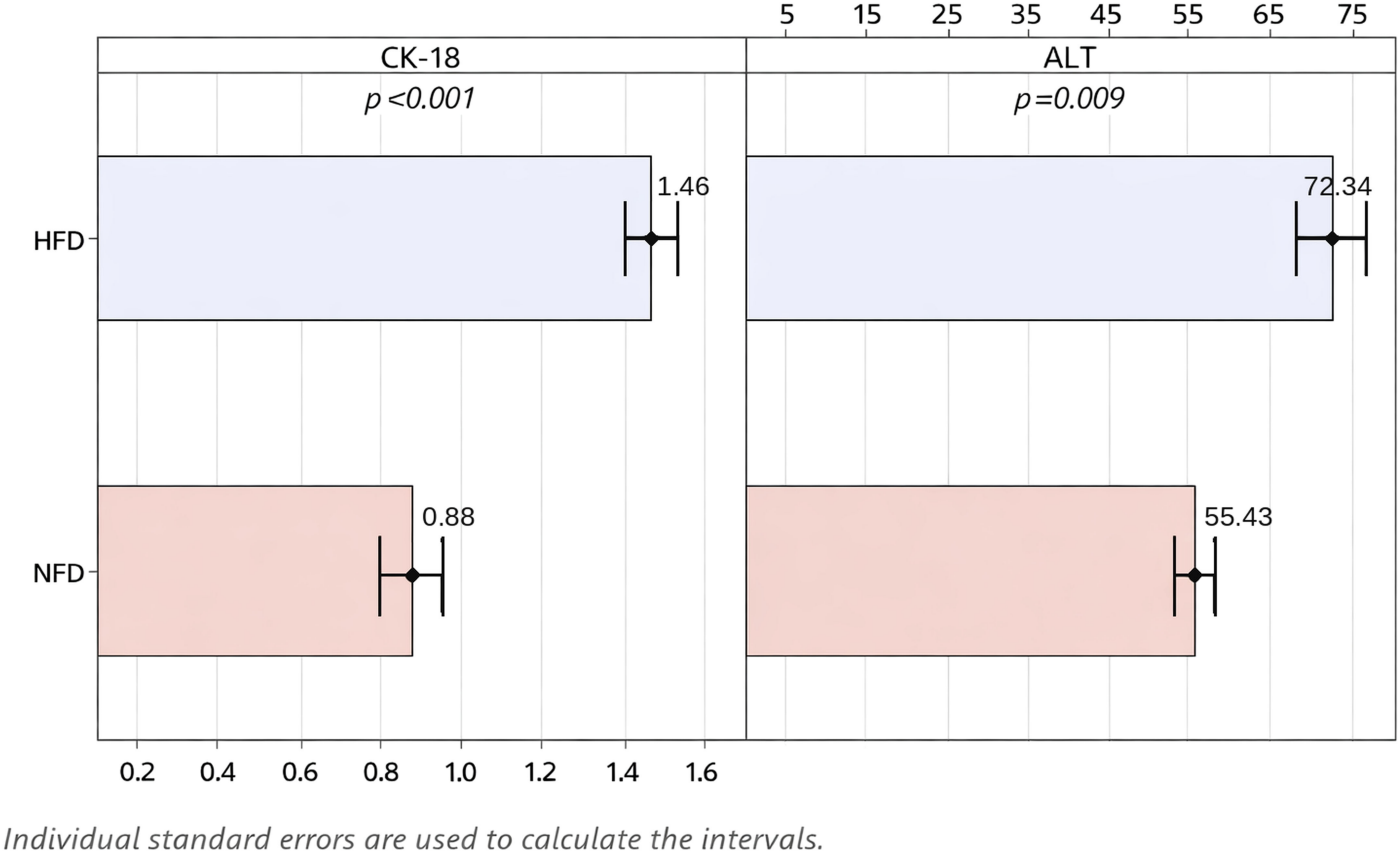
The comparison of CK-18 and ALT levels between the groups.

HFD group significantly increased final body weight compared with NFD group (p<0.001), corresponding to a larger mean weight gain over the 8-week period. Fasting glucose and insulin levels were higher in HFD group than in NFD group (p=0.011 and p<0.001 respectively). Consistent with these findings, HOMA-IR was higher in the HFD group than in the NFD group (2.03 ± 0.79 vs 0.73 ± 0.34; p=0.003). ALT level was higher in HFD group (p=0.009), while AST level showed a nonsignificant increase. AST/ALT ratio was lower in HFD group (p=0.020). Lipid parameters and lipid-derived ratios did not differ significantly between the groups (all p>0.05). The mean difference (HFD–NFD) in CK-18 was 0.59 ng/ml (95% CI 0.38–0.80), corresponding to a large standardized effect (Hedges’ g≈3.07, p<0.001). Morphometric and laboratory parameters of the study were shown in **Table 1**. The comparison of CK-18 and ALT levels between the groups was shown in **Figure 1**.

**Table 1:** Morphometric and laboratory parameters of the study.

Parameters	Groups		p	Hedges’ g (HFD vs NFD)
	NFD (n=6)	HFD (n=8)		
Initial BW (g)	268.00 ± 9.0	270.75 ± 9.30	0.932 ^a^	0.281
Final BW (g)	291.50 ± 6.25	335.8 ± 20.20	**<0.001** ^a^	2.601
Glucose (mg/dL)	130.00 ± 16.19	186.38 ± 46.91	**0.011** ^b^	1.414
Insulin (IU/L)	2.23 ± 0.82	4.30 ± 0.70	**<0.001** ^a^	2.576
HOMA-IR	0.73 ± 0.34	2.03 ± 0.79	0.003^a^	1.90
ALT (U/L)	55.43 ± 5.75	72.34 ± 12.35	**0.009** ^a^	1.562
AST (U/L)	73.03 ± 7.08	83.00 ± 10.18	0.063 ^a^	1.035
AST/ALT	1.32 ± 0.14	1.16 ± 0.09	**0.020** ^a^	-1.319
CHOL (mg/dL)	48.50 ± 11.00	53.38 ± 6.12	0.309 ^a^	0.537
TG (mg/dL)	118.33 ± 38.74	122.88 ± 43.18	0.842 ^a^	0.103
LDL (mg/dL)	10.67 ± 1.86	12.25 ± 2.25	0.188 ^a^	0.706
HDL (mg/dL)	46.67 ± 14.07	62.89 ± 22.15	0.144 ^a^	0.791
CHOL/HDL	1.06 ± 0.11	0.90 ± 0.19	0.096 ^a^	-0.927
TG/HDL	2.79 ± 1.46	2.07 ± 0.86	0.270 ^a^	-0.587
CK-18(ng/mL)	0.88 ± 0.18	1.46 ± 0.18	**<0.001** ^a^	3.07

NFD, Normal-fat diet; HFD, High-fat diet; ALT, Alanine aminotransferase; AST, Aspartate aminotransferase; CHOL, Total cholesterol; TG, Triglyceride; LDL, Low-density cholesterol; HDL, High–density cholesterol; CK-18, Cytokeratin-18. HOMA-IR, Homeostatic Model Assessment for Insulin Resistance.

^a^ Student’s t-test.

^b^ Welch’s t-test.

Positive Hedges’ g values indicate higher values in HFD; negative values indicate lower values in HFD. Conventional interpretation: 0.2 small, 0.5 moderate, 0.8 large.

Bold values indicate statistically significant p-values (p<0.05).

### 3.2 Histopathological results

**Figure 2:**
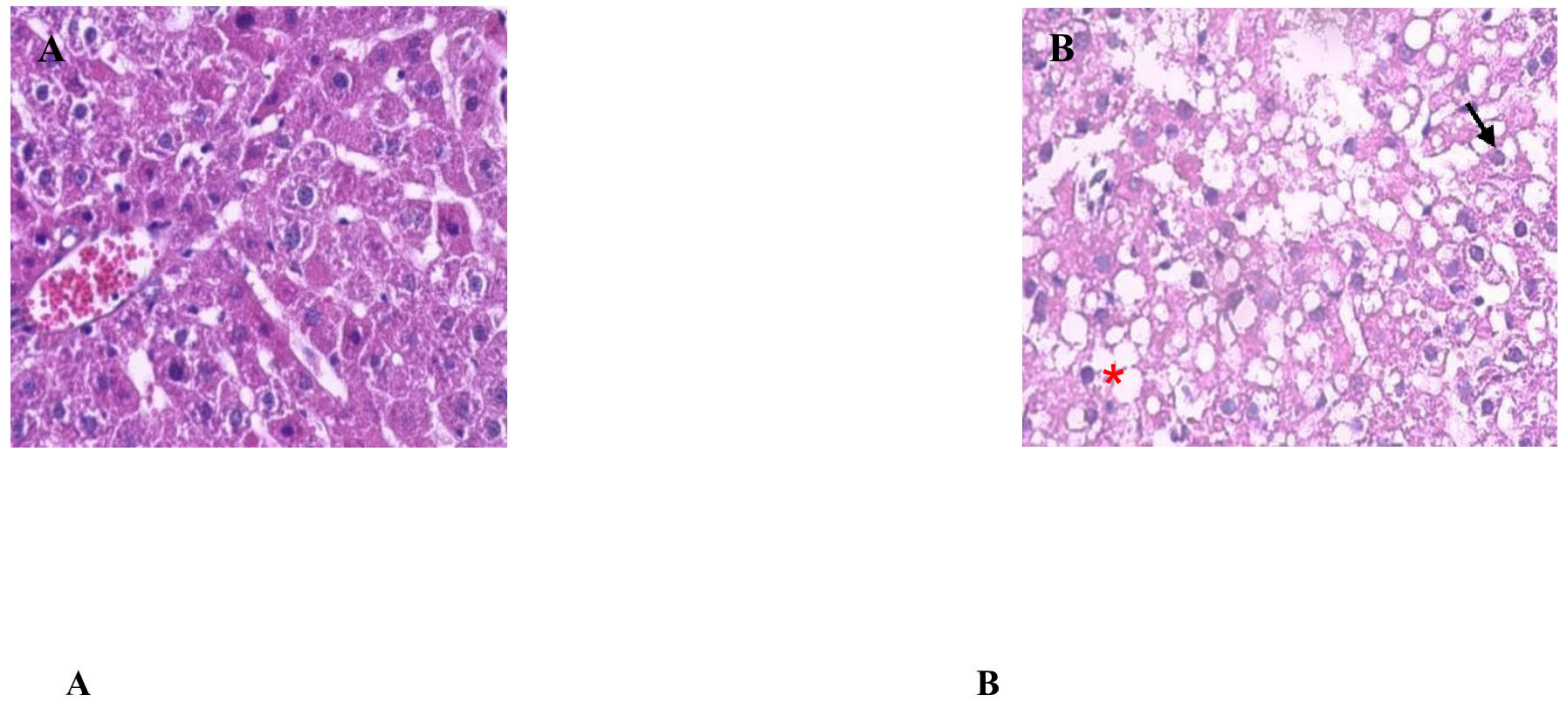
Liver specimens according to the groups. **(A)** NFD, **(B)** HFD.

All NFD liver specimens showed normal architecture without steatosis, ballooning, inflammation, or fibrosis. In the HFD group, hepatocellular ballooning was present in all specimens (8/8) and differed significantly between groups (p<0.001). Steatosis and lobular inflammation were each observed in 4/8 of HFD specimens but did not reach statistical significance in between-group comparisons (both p=0.085). Mild fibrosis (stage 1) was observed in 2/8 specimens and did not differ between groups (p=0.473). Consistent with these findings, NAS was higher in HFD than NFD (median 3.0 [3.0–3.0] vs 0.0 [0.0–0.0]; p=0.001), largely reflecting the uniform ballooning component with additional contributions from steatosis and inflammation. NAS values did not exceed 3 in any specimen, suggesting that histological activity remained below commonly used NAS thresholds for NASH. The histopathological changes in each liver tissue specimen with each CK-18 level of the groups were shown in **Table 2**. The normal liver tissue appearance in NFD group specimens and the liver tissue changes in HFD group specimens, particularly the ballooning of liver cells in HFD group, were clearly observed. The histopathological images of the liver specimens according to the groups were shown in **Figure 2**.

**Table 2:** The histopathological changes and CK-18 levels in liver tissue samples of the groups.

Groups	Specimen	Steatosis	Ballooning	Inflammation	Fibrosis	CK-18
NFD	1	**0**	**0**	**0**	**0**	1.05
	2	**0**	**0**	**0**	**0**	0.64
	3	**0**	**0**	**0**	**0**	0.69
	4	**0**	**0**	**0**	**0**	0.95
	5	**0**	**0**	**0**	**0**	0.86
	6	**0**	**0**	**0**	**0**	1.06
HFD	1	**1**	**2**	**0**	**1**	1.48
	2	**1**	**2**	**0**	**0**	1.69
	3	**0**	**1**	**0**	**0**	1.55
	4	**0**	**2**	**1**	**0**	1.50
	5	**1**	**1**	**1**	**0**	1.11
	6	**1**	**2**	**0**	**1**	1.42
	7	**0**	**2**	**1**	**0**	1.34
	8	**0**	**2**	**1**	**0**	1.62
p		0.085^a^	**<0.001** ^b^	0.085^a^	0.473^a^	**<0.001** ^c^
Effect size		0.548	1.000	0.548	0.354	3.069

NFD, Normal fat diet; HFD, High fat diet.

^a^ Fisher’s exact test.

^b^ Exact Mann–Whitney U test.

^c^ Student’s t-test.

Binary histopathological features are shown as prevalence (n/N) and use φ coefficient as the effect size. Ballooning score uses rank-biserial correlation (r_rb). CK-18 uses Hedges’ g.

Bold values indicate statistically significant p-values (p<0.05).

### 3.3 Correlation among ALT, CK-18 levels and histopathological results

**Figure 3:**
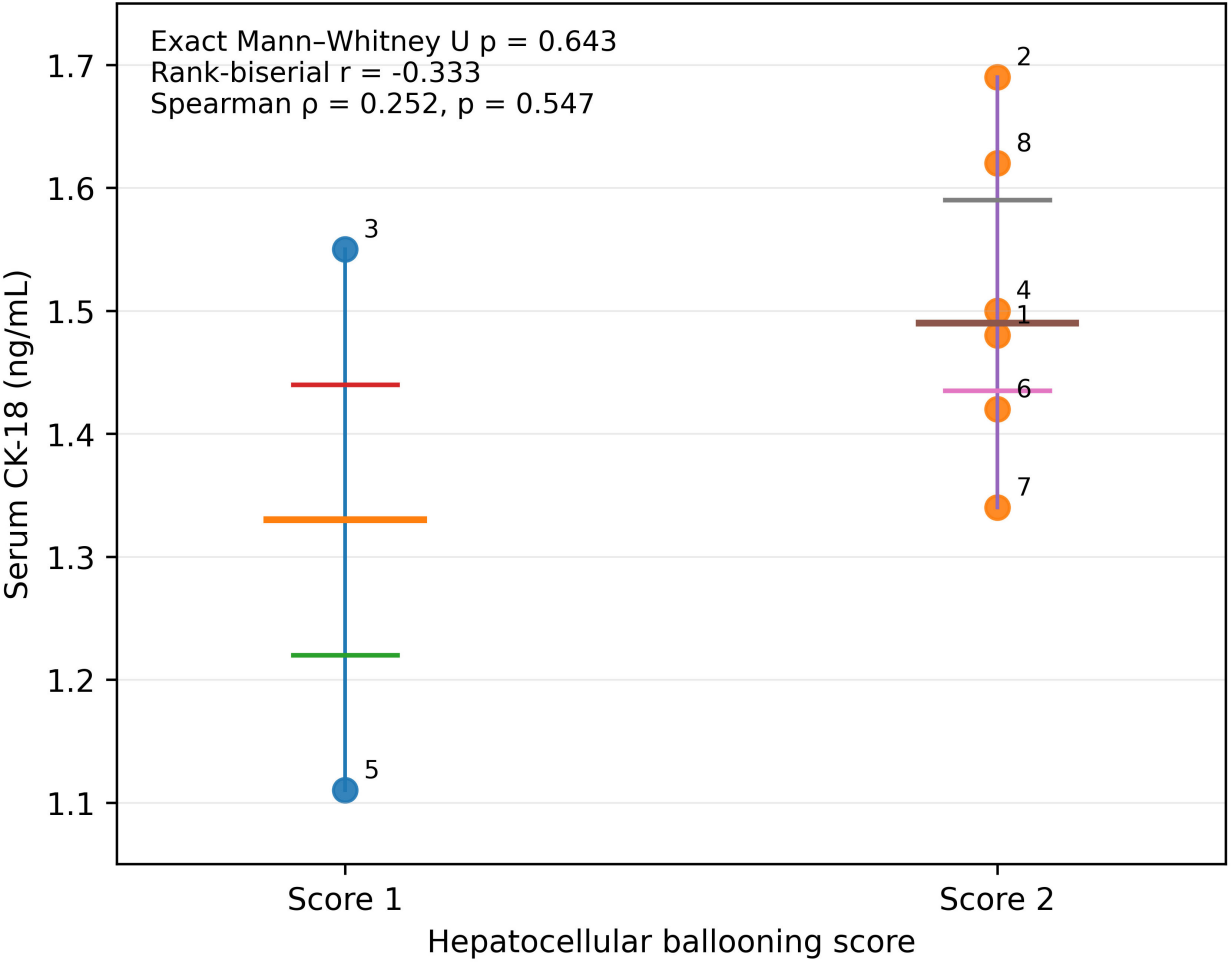
Serum CK-18 levels according to hepatocellular ballooning score in HFD group.

Although ALT and CK-18 levels were both higher in HFD group than in NFD group, this between-group pattern did not indicate a positive within HFD association among ALT, CK-18 and ballooning. CK-18 did not differ significantly between the specimens with ballooning score 1 and those with ballooning score 2 (exact Mann–Whitney U, p=0.643), and the association between CK-18 and ballooning score was weak (Spearman’s ρ=0.252, p=0.547) within the HFD group. The distribution of CK-18 levels according to ballooning severity was illustrated in **Figure 3**.

## 4 Discussion

In the present study, with high-fat cafeteria diet model, we observed the increased weight gain, hyperglycemia, hyperinsulinemia, and elevated ALT and alongside increase in circulating CK-18. Angulo and Lindor found that NAFLD developed in approximately 94% of obese individuals, 67% of overweight individuals and 25% of normal individuals (20). Because of its close association with obesity, insulin resistance, and metabolic disorders, NAFLD has been referred to as MASLD in recent years. The most clinically used screening tests to assess MASLD, are transaminase values. The increase of ALT and AST levels are still using for NAFLD diagnosis in first step (21). In the present study, ALT level was significantly increased with HFD but AST did not change to a statistically significant level. However, Torres et al. stated that liver enzymes may be normal in up to 78% of patients with NAFLD (22). Similarly, Mofrad et al. demonstrated that the patients with normal ALT levels may have NAFLD with characteristic histopathology (23). Kunde et al. showed that ALT has only 40% of the diagnostic accuracy for NASH assessment (24). On the other hand, Obika et al. stated that ALT and AST do not always increase in the fatty liver, nor do they have no marker property for any stage of NAFLD (25). In recent years, lipid-related indices were questioned in terms of the association with NAFLD. In one study, an association between TG/HDL-C and NAFLD has been reported, and it was stated that this parameter may be useful for the NAFLD risk screening in the general population (26). Another study demonstrated that higher TG/HDL-C ratio was associated with NAFLD and metabolic syndrome (27). In the present study, lipid-related indices of NFD and HFD groups were not found statistically different. Anyway, elevated lipid levels are not directly related to NAFLD; rather, intracellular fat accumulation within liver cells constitutes its pathogenesis. This initial stage may progress accompanied by inflammation and apoptosis. In the present study, a significant increase of CK-18 level was observed with the increase of body weight, glucose, insulin and ALT levels. Some of the studies suggested that CK-18 has a positive correlation with serum ALT levels in NAFLD however, the guidelines for the prevention and treatment of non-alcoholic fatty liver disease, reported that elevated levels of serum ALT and CK-18 need to be confirmed by liver biopsy results for the diagnosis NAFLD severity (28, 29). A meta-analysis reported that the many studies noted different non-invasive markers with their different characteristics and advantages, but also emphasized.the benefit of evaluating them all together for NAFLD (30). Feldstein et al. found that serum CK-18 levels correlated with both the degree of steatosis and the degree of fibrosis in NAFLD. They stated that CK-18 can differentiate between moderate and severe fibrosis, especially in progressive cases such as NASH (17). Wu et al. concluded that serum CK-18 can predict the stage of NASH in NAFLD similarly, Kawanaka et al. reported that CK-18 is a predictive marker for the prognosis of NAFLD (31, 32). In the study of Younossi et al. cytokeratin CK-18 (M65 antigen, a measurement of overall cell death due to both apoptosis and necrosis) and caspase-cleaved CK-18 (M30 antigen, a specific measurement of apoptosis) were profiled. Both M30 and M65 biomarkers were found higher in NASH and related fibrosis (33). However, Cusi et al. suggested that plasma CK-18 has insufficient power to screen the NASH stage because of its limited sensitivity, despite its high specificity for NAFLD and fibrosis (34). CK-18 is a major intermediate filament protein which constitutes approximately 5% of liver proteins in hepatocytes. The release of CK-18 fragments occurs at the onset of cell death, so its level is more associated with hepatocyte apoptosis (35). In the present study, we measured total circulating CK-18 which does not isolate the apoptosis-specific caspase-cleaved fragment the M30 neoepitope from the M65 epitope. Therefore, the CK-18 increase should be read as a marker of overall hepatocyte death in our study.

The main finding of the present study is that CK-18 levels were higher in all rats of the HFD group compared to those of the NFD group. When the pathological samples of the HFD group were examined, even with the absence of NASH status, inflammation was observed in 50% of the samples and fibrosis in 25% of the samples. However, ballooning was observed in 100% of the samples. This was remarkable. When we planned thıs study, our prımary aım was to observe what changes occur ın lıver cells, and also to examıne CK-18 levels, as obesıty begıns to develop. Wıth our results, we do not claım that CK18 and lıver cell balloonıng are dırectly lınked. However, we want to draw attentıon to the fact that they both changed as obesıty begıned. The ballooning disorder can be associated with the obesity effect on liver cells. In one study, it was observed that hepatocyte swelling and alterations in cytoskeleton proteins developed with obesity and metabolic dysregulation, This damage result in the rupture of intermediate filaments and the fragments of CK-18 release into the peripheral blood (36). In obesity, lipid accumulation and the burden of the inflammatory state and oxidative stress in liver cells, histopathological changes, and progressive apoptosis determine the development and severity status. A study examining the pathological development showed that both simple steatosis and NASH have the characteristic histological finding as “hepatocyte ballooning” (37). This is due to the accumulated fat pushing the nucleus to the periphery and increases cell size to approximately 1.5 to 2 times the normal size (38). The balloning is one of the major changes in the development of the NASH stage and has reported previously as an increasing effect on fibrosis (39). One of the recent studies investigating the predictive value of CK-18 level in patients with NAFLD showed that CK-18 level changed with the hepatocyte ballooning grades but not with the fibrosis stages (40). Again, a recent study stated that CK-18 was highly correlated with NASH and could be also used to diagnose other stages of NAFLD (41). Looking at the studies with differing results in the literature, it is known that there is no consensus on whether CK-18 is a marker for a specific histopathological stage in NAFLD. In the present study, the histopathological injury in the HFD group was characterized predominantly by uniform hepatocellular ballooning, with additional contributions from steatosis and lobular inflammation. Overall, these findings indicate early hepatocellular injury without overt steatohepatitis. In addition, the change of metabolic parameters and CK-18 increase were also observed. Detecting the presence of injury (CK-18 differed strongly between groups) and grading its severity (a within-group gradient) are distinct questions, and the present study addresses the former. Accordingly, CK-18 is best regarded here as a signal of the presence of early hepatocellular injury rather than a measure of its histological grade.

The terms NAFLD/NASH were used in our study for the sake of literature consistency, hovewer, our findings are directly related to current MASLD/MASH guidelines. Obesity or excess weight is the primary of the metabolic risk factors identified for a direct diagnosis of MASLD. This change in nomenclature moves away from an exclusionary definition based solely on alcohol and centers on direct metabolic dysfunction criteria such as obesity. The term MASH is a more emphatic expression to clarify the link between metabolic dysfunction and the risk of inflammation and fibrosis in the liver. The liver cell changes presented with the development of obesity in our study fully associated with metabolic changes.

Non-invasive identification and follow up of liver damage in obese patients and the association with CK-18 levels were the subject of recent researches. In one study, it was reported that CK-18 level may reflect liver damage and the complications of obesity such as insulin resistance and disturbed lipid metabolism (42). In another study, CK-18 level above the median values showed significantly higher waist circumference, HbA1c, AST, ALT, HoMA-IR, compared to those with values below the median. It was reported that CK-18 levels correlated with anthropometric and metabolic parameters representing the visceral obesity (43). One of the recent studies was performed in patients with obesity undergoing laparoscopic sleeeve gastrectomy. The parameters body mass index, insulin resistance, lipid parameters, ghrelin, leptin, AST/ALT ratio, and CK-18 levels ​​improved after the surgery period. Serum CK-18 was found more predictive than other biomarkers in monitoring **MASLD** with obesity (44).

### 4.1 Limitations

This study has some limitations. This is an experimental study, and it cannot provide a clinical target. Although the group sizes were acceptable for the experimental rat model, the death of two NFD rats reduced the final control group size and may have limited statistical power for between-group comparisons. The number of rats was limited for within-group correlation analyzes and subtle relationships. A definitive conclusion regarding the relationship between ballooning degrees and CK-18 levels cannot be drawn. We measured total circulating CK-18 and our data cannot establish the mode of cell death. Paired M30 and M65 measurements are needed to define the contributions of apoptosis and necrosis.

## 5 Conclusion

Our results indicate that high-fat-diet–induced obesity was associated with hepatocellular ballooning and with increased CK-18 levels before the development of overt steatohepatitis These findings should be interpreted as associative rather than causal. Further studies interpreting CK-18 and liver cell changes with a larger number of cases on obesity may give more knowledge on their relationships.

## Data Availability

The original contributions presented in the study are included in the article/supplementary material. Further inquiries can be directed to the corresponding author.
